# Opinions of Teachers on Distance Education Applications in English Language Teaching Policies in Northern Cyprus During the COVID-19 Pandemic

**DOI:** 10.3389/fpsyg.2022.868198

**Published:** 2022-04-28

**Authors:** Merve Uysal, Çağda Kıvanç Çağanağa

**Affiliations:** Department of Educational Adminisration and Supervision, European University of Lefke, Lefka, Turkey

**Keywords:** Northern Cyprus, COVID-19, distance education, foreign language education policy, foreign language teaching

## Abstract

The COVID-19 pandemic affected education institutions just like several other sectors. These institutions, which are among the places where people are found collectively, were the first places that were closed for precaution. Some problems of distance education conducted in online platforms which were assumed upon the termination of face-to-face education emerged in time. Especially in this urgent solution, where the workload of teachers has increased, some application difficulties have been identified in foreign language teaching. From this point of view, considering the foreign language education policy and planning, this study deals with the distance education practices of English teachers, the difficulties they encounter in the process, and their solution proposals. The research, which was designed as a qualitative study, was conducted with a case study design. The participants of the research are 13 English teachers working at the secondary school level in different districts of Northern Cyprus determined by the homogeneous sampling method. The data were collected through semi-structured interviews developed by the researchers. The qualitative data were analyzed with descriptive analysis. As a result of the interviews with the teachers, it has been determined that there are problems related to technology, students, online classes, and planning in English teaching carried out with distance education applications. Accordingly, the teachers offered their recommendations that could be a solution to the existing problems.

## Introduction

Language policy is of critical ethical, political and legal importance to all authorities around the world. Spolsky (2004) argues that language policy is a product of related traditions, beliefs and preferences in the society although they may seem independent from each other. However, most language policy decisions made in a country depend on the bureaucracy. They are taken by government agencies and the practice is based on regulations made by bureaucratic authorities (Bamgbose, 2020). In countries where English is taught as a foreign language, foreign language education policy plays a critical role. Foreign language education policy is defined as “a type of language policy” (Poon, 2000) concerned with the organization of language teaching within the formal education system that aims to increase students’ proficiency in their mother tongue or to add new languages to their repertoire through teaching a second or foreign language. Therefore, it has been developed to meet the foreign language needs of a country and to address issues related to foreign language education in school settings.

Foreign language education policy is related to all dimensions of education, from national scale to classroom scale and from primary education level to university and adult education (Hult, 2018). Kaplan and Baldauf (1997) explained that language planning in education includes curricula and related decisions informed by federal, local and state policies. In addition, he stated that the policy in question covers training, development and professional practice of foreign language teachers, creation and implementation of teaching materials informed by research-based methodologies, and evaluation of students, schools and policies. Language policies in education are formed by the efforts of official bodies such as education ministries in language planning; this initiative sets the goals and means for the implementation of language policies, which establish the rules or guidelines that shape the learning, use and structure of language in educational institutions (Tollefson, 2008).

Baldauf (2006) emphasizes that language policy includes ideas, laws, regulations, rules and practices aimed at planned language change. This policy can be implemented through language planning documents. Baldauf claims that language policy and planning occur at different levels, and he recognizes a macro (national) level that refers to the political process at the top and a micro (local) level that refers to the process of implementation at the bottom. Likewise, Spolsky (2004) argues that language policy can take place in two levels, namely macro and micro level. Accordingly, examples of micro level in sociolinguistic contexts within language policy can be families, schools, religious organizations, agencies and local governments, while international organizations and policies constitute examples of macro level. Byrnes (2003) argues that several countries in the world have adopted a centralized approach to language policy making. Shohamy (2006) states that decisions on foreign language education policy are made by central authorities such as government agencies, parliaments, ministries of education, regional education boards and schools. Since political, social and economic factors are in close contact with each other, they are interdependent in many aspects. In addition, in the context of foreign language education policy, globalization process and the Council of Europe have a considerable impact on the formation of the content of the said policy. Due to globalization and technological innovations, foreign language education policies are affected by the worldwide popularization of English. The Council of Europe, on the other hand, helps the states that are members of the Union to carry out parallel foreign language education policies and practices. Therefore, foreign language education policy, which is shaped by several different variables, is put into practice by political authorities from top to bottom. Schools and teachers comply with these policies. As a result, they are blended with teachers, teaching materials, curricula and assessment-evaluation methods.

Several countries around the world take a strict top-down approach to language policy planning. As a matter of fact, this type of planning, which is designed with an inflexible top-down approach, has to be disrupted in some cases which makes it hard to predict the results. The most obvious example of this situation is the Coronavirus pandemic, which emerged in the form of an epidemic in Wuhan, China in December 2019 and continues its effect by spreading globally. In such periods, when the normal process has to be bypassed, effort is paid to develop a quick solution. In this case, it was distance education applications which were introduced in order to prevent the interruption of education. In times of such uncertainty and crisis, it is very difficult to make long-term plans and implement them (Uysal and Karagöz, 2021). Several researchers agree that education systems that have developed in the last 50 years have not faced such a complex challenge (Daniel, 2020). The COVID-19 global pandemic is, in fact, an unforeseen emergency crisis situation and therefore decisions for the solution have to be taken fast. The first measure taken as regards education in all countries was the closure of schools and the interruption of education. However, as the effect of the pandemic spread, it was decided that taking a brief break from education was not enough, and practices were adopted to continue education on distance education platforms (Serçemeli and Kurnaz, 2020).

Distance education is an education model in which the learner makes progress according to his/her own learning style and pattern, regardless of time and place (Uysal and Karagöz, 2021). Distance education, which is applied during the pandemic, is the temporary transfer of traditional education to online platforms in a crisis; hence itis called “emergency distance education.” This teaching method includes the use of distance education solutions in crisis or emergency situations where education and training activities cannot be carried out face to face. Curriculum and course materials prepared for traditional education are transferred to online environments, which is preferred as a solution for emergencies. In this respect, emergency distance education and distance education are distinguished from each other (Akkoyunlu and Bardakçı, 2020).

Similar to the case in almost all countries, after the first case was seen in Northern Cyprus on March 10, 2020, all schools from basic education to higher education institutions switched to the distance education system, and it was believed that this system might continue in the ongoing period. After returning to traditional education, schools on the island had to reassume distance education system frequently due to the fluctuations in case statistics. In this case, teachers try to conduct their classes through various applications, especially Microsoft Teams, Google Classroom, Zoom, and Whatsapp. Due to the emergency situation at the very beginning of this process, most of the students started online education without any readiness, and most teachers tried to teach in the distance education system despite their lack of online teaching experience.

Especially in this process where online technologies are indispensable due to the COVID-19 global pandemic, language or foreign language teaching practices, which represent a unique learning area unlike other courses taught in schools, have also taken their share from this adaptation process. Various studies show that foreign language learning can benefit from the distance education system (Tümen-Akyıldız, 2020). The active use of technology in foreign language learning helps students gain more familiarity with the target language and allows them to learn the language in its real environment. Through technology, people find social interaction opportunities that they cannot find inside or outside the classroom, and they can continue their activities in coordination with other students based on real-life inspirations (Parvin and Salam, 2015). For example, there are studies showing that Whatsapp, one of the most widely used mobile phone applications, increases the efficiency of foreign language teaching (Jasrial, 2018). In a study conducted by Alsaleem (2021), it was concluded that with the use of Whatsapp application in foreign language teaching, students improved their speaking, writing and vocabulary. In another study, it was observed that the Whatsapp application used during foreign language learning increased students’ speaking skills and learning motivation (Bahruddin and Febriani, 2020). Teaching online has both positive and negative consequences. The most obvious of the negative outcomes is observed when students do not have the necessary technical equipment used in the distance education system (Aliyyah et al., 2020). In his study, Tonbuloğlu (2017) concluded that distance education system reduces the productivity of the students when the course teaching program is not suitable for the virtual environment, different learning methods cannot be offered, and interaction is missing. In addition, it has been revealed that teachers have difficulties in measuring English reading and listening skills of students in the online environment (Nilsson, 2021).

Although the realization of foreign language teaching in the virtual environment dates back to before COVID-19, the emergency distance learning process applied during the pandemic period is much more complicated. It is not surprising that all the activities of the face-to-face English course curriculum, which was planned at the macro level due to the internal dimensions and challenging conditions of the global pandemic, were not carried out. Moreover, the speed and success of the formulation and implementation of education policy in times of crisis is closely related to the state infrastructure. As the COVID-19 pandemic spread rapidly on the Island, the Turkish Cypriot education system did not have time to prepare for the possible effects of digital distance education. Nevertheless, the extent to which the problems experienced in foreign language teaching are related to the new practices remains unclear. When the English course is taken into consideration, it can be said that the transition from the education that is traditionally planned face-to-face to the online environment has caused some problems for both students and teachers. While the uncertainties (according to these conditions) that started due to the COVID-19 pandemic and still continue with the deaths and cases on the Island point out that the emergency distance education process will continue for a while, it is also clear that teachers who have to continue their foreign language teaching in this period have a lot of work to do. Therefore, it is critical for new distance education activities that English teachers deliberate on the process and come up with some solutions to deal with the problems they encounter. A review of the literature review shows that there are few studies on the opinions of teachers about teaching English during the COVID-19 global pandemic (Tümen-Akyıldız, 2020; Yi and Jang, 2020; Cantürk and Cantürk, 2021). To the best of the researcher’s knowledge in the context of Northern Cyprus, it was concluded that there are no studies that involve the experiences and suggestions of English teachers. In this respect, it is anticipated that the study will fill a gap in the literature and serve as a roadmap for English teachers and policy makers.

From this point of view, the current research aimed to reveal the opinions of English teachers regarding the distance education applications which were adopted as an alternative to the face-to-face education system during the pandemic process in Northern Cyprus. The purpose of the study is to display the difficulties they encountered and the solution proposals they developed for these problems in terms of planning of foreign language education policy. In order to serve these purposes, the research questions were formed as follows:

1. What are the distance education activities carried out by English teachers within the scope of distance education?
2. What are the difficulties that English teachers face in their distance education activities within the scope of distance education?
3. What are the solutions of English teachers for the future distance education activities?

## Method

### Research Model

This research is a case study which employs a qualitative research model. A case study is a model in which the researcher interprets themes related to situations by examining one or more cases in depth from different sources in a certain period of time (Creswell, 2007). The purpose of choosing a case study is to enable the researcher to obtain in-depth information about a current phenomenon from different sources.

### Study Group

The research group of the study consists of English teachers who teach at secondary school level in different public schools in Northern Cyprus using emergency distance education applications in the 2021–2022 academic fall semester. According to the Education Statistical Yearbook published by the ministry in 2020, 500 English teachers are working in different public secondary schools (Ministry of National Education and Culture [MONEC], 2020). Therefore, in order to explain the subject studied in depth, 13 participants determined by using homogeneous sampling method were included in the research. The purpose of this sampling method is to select small and peer samples in order to work with subgroups with certain common denominators (Neuman, 2014). The participants of the research are English teachers who have experienced distance education applications using different online platforms. Personal information about the participants is presented in Table 1 below.

**TABLE 1:** Demographic information of participants.

Gender:	Number (13)	Male:3	Female:10	
Level of education:	Number (13)	Undergraduate:7	Postgraduate:6	PhD: -
Professional seniority:	Number (13)	0–10 years:1	11–20 years:6	21 years and above: 6

Considering the information in Table 1, ten of the teachers are females and three are males. The education level of the teachers participating in the research is as follows: 7 of them have a bachelor’s degree and 6 participants have a master’s degree. Finally, looking at the professional seniority of the teachers, it was determined that 1 teacher worked for 0 to 10 years, 6 teachers worked for 11 to 20 years, and 6 teachers worked for 21 years or more.

### Data Collection Tool

Semi-structured interviews were conducted in accordance with the purpose of the research. Dörnyei (2007) explains that the semi-structured interviews are useful because their format is open-ended and the interviewer is given freedom to express his/her ideas in depth. Therefore, semi-structured interview technique was preferred in order to provide in-depth data on the research questions. In this way, a form developed by the research consisting of two parts was used as a data collection tool. Interview questions were prepared after reviewing the relevant literature. The created form was presented to two Turkish language academicians specialized in content validity, language and expression. As a result of the feedback received from these specialists, the interview form was reformatted and finalized with minor changes made on the questions. Accordingly, in the first part, which also includes the informed consent form, the demographic information form (gender, education level, and professional seniority) created to identify the personal information of English teachers was used. The second part includes four open-ended questions. The interviews with the teachers took place between 10.12.2021 and 30.12.2021 in the virtual environment.

### Analysis of Data

Descriptive analysis technique was adopted in the analysis of the data collected through the interviews. In the descriptive analysis technique, data can be interpreted through pre-built themes. The main purpose of this type of analysis is to present the findings as interpreted by removing the summary points (Dawson, 2009). During the fieldwork, researchers collected the data in Turkish, translated them into English, transcribed them, and coded them. Thus, each interview was presented to the readers based on its original form. In order to ensure validity and reliability, the data were coded by a field expert other than the researcher, and a consensus of over 70% was reached according to the reliability formula of Miles and Huberman (1994). While giving examples of the opinions of English teachers in the research, teachers were given codes such as T1, T2, … and T13.

## Findings

Findings reached within the scope of the research are presented in tables following each research question.

### Findings Related to the First Research Question

As can be seen in Table 2, the distance education activities carried out by the teachers were grouped under 4 themes for the first research question. These themes were named as “activity and document sharing,” “lecturing,” “homework and project control” and “collaboration with stakeholders.”

**TABLE 2:** Distance education activities of teachers.

Theme	Codes	F
Activity and document sharing	Document sharing through e-mail and Whatsapp application	T1, T2, T3, T4, T5, T6, T7, T8, T9, T10, T11, T12, and T13
	Video sharing with lecture content	T1, T4, T5, T7, T8, T10, T11, and T13
	Online exam sharing	T2, T3, T4, T6, T7, T8, T9, and T13
	Interactive exercises	T1, T2, T4, T6, T8, and T9
	Practices on four basic skills	T5, T6, T10, T11, and T12
Lecturing	Online lectures	T1, T2, T3, T4, T5, T6, T7, T8, T9, T10, T11, T12, and T13
Homework and project control	Homework-project control through e-mail	T1, T2, T3, T4, T5, T6, T7, T8, T9, T10, T11, T12, and T13
	Individual homework-project control through virtual platforms	T3, T4, T6, T8, T9, T10, T11, and T13
Collaboration with shareholders	Communication with parents	T1, T4, T5, T6, T7, T11, and T13
	Individual meeting with students	T3, T6, T9, and T10

Document sharing by all of the teachers *(f* = *13) via* e-mail and WhatsApp application formed the first code under the theme of “activity and document sharing.” T7 commented: “*I sent homework and activities to the children via WhatsApp. Sometimes I gave homework before the online class started, and sometimes after the class*.” T9 made the following comment: “*I sent homework to my classes via the e-mail group I created, and I always sent worksheets.*” The fact that most of the teachers *(f* = *8)* shared course videos with their students formed the second code of this category. T13 said: “*During the class, I opened videos on YouTube and then sometimes I sent videos and links to students to reinforce the subject*.” Based on the necessity of making an exam system fit for distance education applications, the fact that the majority of teachers *(f* = *8)* shared online exams created the third code. T4 explained the situation as follows: “*Since we delivered the classes online, we also performed the exams online via Google forms; after all, the results were taken automatically, which was pretty good. The results of the exam gave the opportunity to discuss them immediately*.” As the fourth code, the participants *(f* = *6)* stated that they tried to include interactive exercises in virtual classrooms. T2 commented: “*I try to send interactive worksheets during the class from the Zoom*.” T8 made the following comment: “*I don’t just teach the class, I also ask questions to the children so that they are always engaged*.” The last code consists of participants *(f* = *5)* who try to ensure that all four basic language skills are given equally in foreign language teaching. T5 expressed the importance of these skills in the scope of the English class curriculum: “*I don’t just focus on monotonous grammar because we can do virtual classes in practice, whether it’s writing, reading, speaking, listening.”*

The second theme is that all participants *(f* = *13)* deliver online lectures through Google Classroom, Microsoft Teams and Zoom virtual classes in distance education. T13 said: “*I continued my classes remotely by sending links to my children via WhatsApp*.” The third theme is homework and project control. The fact that all of the teachers *(f* = *13)* checked the homeworks and projects *via* e-mail formed the first code of the theme. T7 said: “*I checked by email to see if any of the students in the class had done their homework or projects. Sometimes there was a problem in the system, and e-mail seemed more secure to me*.” In addition, it was observed that some of the participants *(f* = *8)* performed homework and project control using virtual platforms. T3 stated as follows: “*It was much easier to check and assign homework through the platform I used because I could give immediate feedback*.”

The last theme is cooperation with stakeholders. The fact that the participants *(f* = *7)* were in contact with the parents formed the first code of the theme. T4 reported: “*When students are not attending the class, I usually contact the parents and ask them to provide support in this regard*.” The fact that some of the teachers *(f* = *4)* had one-on-one meetings with the students formed the second code of this theme. T9 stated that they tried to support the students as follows: “*I had a one-on-one meeting with the student who I thought did not understand the subject. I always tried to be in contact with students either via WhatsApp or e-mail*.”

### Findings Related to the Second Research Question

In Table 3, the difficulties faced by teachers in their distance education activities revealed 4 themes for the second research question. These themes were classified as “technological infrastructure problems,” “student-based issues, “problems encountered in online courses” and “problems related to planning.”

**TABLE 3:** Difficulties faced by teachers in distance education activities.

Theme	Codes	F
Technological infrastructure problems	Internet shortage	T1, T3, T4, T5, T6, T7, T8, T11, T12, and T13
	Lack of hardware	T2, T3, T4, T5, T7, T8, T9, T10, and T13
Student-based issues	Low participation in class	T4, T5, T6, T9, T10, T11, T12, and T13
	Indifference to the class	T2, T7, T8, T13, and T7
	Low motivation	T1, T3, and T4
Problems encountered in online course	Students not turning on their cameras	T3, T5, T6, T7, T8, T9, T10, T11, T12, and T13
	Discipline issues	T1, T2, T3, T7, T9, and T12
Problems with planning	Overcrowded classrooms	T1, T3, T5, T6, T7, T8, T9, T11, and T13
	Inequality of opportunity	T2, T4, T7, T8, and T12
	Limited time	T6, T9, T10, and T11

Internet shortage pointed out by the majority of the teachers *(f* = *10)* formed the first code under the theme of “technological infrastructure problems.” T1 commented as follows: “*Because our country’s internet infrastructure is very slow, there were disconnections, and this is a big problem for us as well. Therefore, it is necessary to develop the infrastructure*.” T11 expressed his discomfort with the situation as follows: “*During the pandemic period, poor internet connections sometimes caused problems.*” The lack of hardware *(f* = *9)* is the second code under the theme. T2 said: “*I think the biggest problem caused by the system is the lack of devices. We have children who do not have computers or tablets. Or not every child can have a computer because there is more than one student living at home. This affected participation in the course.*”

In the second theme titled “Student-based problems,” the code of students’ low participation in the classes is the most frequently mentioned problem by the teachers *(f* = *8).* T4 commented: “*Many students have very low participation because the class is online. Since the parents could not follow, the rate dropped considerably*.” The second code was the discomfort that some of the participants *(f* = *5)* felt because of the students’ indifference to the class. T12 expressed his discomfort as follows: “*Many of the students were very indifferent to the classes compared to face-to-face education. Since I can’t make eye contact while teaching and I can’t observe students one-on-one, their interest is quickly distracted*.” T4, on the other hand, made a comment describing his own distress: “*On the one hand, there are students who do what is given immediately; on the other hand, uninterested students. This situation both hinders the flow of the class and inhibits the enthusiasm of the interested student.*” The third code is the low motivation of students according to some teachers *(f* = *3).* T1 said: “*Students have very low level of motivation, which I think is partly due to the nature of the process.*” T3 explained the situation as follows: “*They are generally not motivated, I have students that I know are successful in the traditional method; but even these students are not motivated anymore.*”

“Problems encountered in online classes” constituted the third theme. The code on which the teachers *(f* = *10)* commented the most was that the students did not turn on their cameras. T12 explained his comment as follows: “*It is a major problem for students not to turn on their cameras. I have to warn them all the time. After all, the cameras are off and I can’t see what the student is doing.*” Disciplinary problems *(f* = *6)* formed the second code of this theme. T2 said: “*There are a lot of problems with classroom management when teaching in a virtual environment*.” T9 emphasized his discomfort with the situation: “*Sometimes during the class, students share sounds and emojis that are irrelevant to the class, or they forget the microphone on and yell. These behaviors, which interrupt the flow, disturb the peace of the class*.” The last theme is “problems with planning.” The code that teachers focused on the most *(f* = *9)* within the scope of this theme was overcrowded classrooms. T7 commented: “*Due to the large number of students, it is very, very challenging to teach four skills. Speaking, for example, which is even difficult in a normal face-to-face classroom environment.*” T11 made the following comment: “*The large number of students causes problems.*” Inequality of opportunity *(f* = *5)* was determined as the second code under this theme. T4 said: “*The fact that not every student has the technical equipment, but also that every teacher does not have enough knowledge, has caused inequalities*.” The fact that some of the teachers *(f* = *4)* had to squeeze their coverage in a limited time formed the final code of this theme. T6 commented: “*It was a problem to finish a class in 30 min in front of the screen that should have taken 4 h.*” Likewise, T10 said: “*The limited time challenges me in terms of the curriculum that I need to cover*.”

### Findings Related to the Third Research Question

When Table 4 is examined, it is seen that the solution proposals for the problems faced by the teachers in the distance education activities they carry out are grouped under 3 themes for the third research question. These themes are listed as “recommendations for technological infrastructure,” “recommendations for the process” and “recommendations for planning.”

**TABLE 4:** Teachers’ solution recommendations for distance education activities.

Theme	Codes	F
Recommendations for technological infrastructure	Unlimited technology and internet support	T1, T2, T6, T7, T9, T10, T11, and T12
	Making up for hardware deficiency	T3, T4, T5, T8, and T13
Recommendations for the process	Effective assessment and evaluation	T2, T5, T6, T7, T8, T9, T11, T12, and T13
	Switching to hybrid education	T1, T3, T4, T8, T10, and T11
Recommendations for planning	Distance education seminars	T1, T2, T4, T5, T8, T9, and T10
	Creating a data pool	T3, T6, T7, T11, and T12
	Determination of course contents by teachers	T2, T10

Unlimited technology and internet support *(f* = *8)* was the first code under the theme of “recommendations for technological infrastructure” indicated by the majority of teachers. T1 said: “*Some of the students had problems especially accessing the internet, or they experienced interruptions in the internet because several people were connected at the same time in the same house. An internet network can be created for students and teachers so that they can access the internet from anywhere.*” T7 expressed his suggestion on this subject as follows: “*Agreement should be made with different telephone operators and unlimited free internet support should be given to the teachers and students.*” T8, one of the teachers who underlined the importance of dealing with the lack of hardware, which is the second code, made the following suggestion: “*I think that technological devices should be provided to students in need by the government*.”

The first code highlighted by the teachers under the theme of “recommendations for the process” is the suggestion of effective assessment and evaluation *(f* = *9).* T12 made the following comment: “*One of the subjects I had the most difficulty with in online education was measurement and evaluation. I wasn’t quite sure if the students were doing the homework on their own or with their friends at that moment. In my opinion, a general evaluation should be made by conducting a coordinated central examination by the Ministry*.” The second code that teachers *(f* = *6)* focused on within the scope of this theme was the transition to hybrid education. T1 made the following comment: “*I think that distance education in the pandemic is very inefficient. In this period, for example, we switched to face-to-face education, but due to increasing cases or contacted teachers and students, we had to switch back to online classes. It becomes even more inefficient this way. I think we should provide direct hybrid education until the end of the process*.” T8 said: “*A hybrid education plan should be made until the end of the pandemic. One day we go to school, then 1 week the class is closed due to contacts and education continues online. But since the content is prepared face-to-face, problems occur; thus, standardization must be ensured*.”

The “recommendations for planning” expressed by the teachers constituted the last theme. In this theme, most of the participants *(f* = *7)* drew attention to the importance of distance education seminars. T5 commented: “*There are differences in the skills and competencies of technology-usage between teachers aged 25 and teachers over 50 years old. In order to eliminate these differences, teachers who feel the need should be supported in terms of technology usage. Seminars should be offered.”* T8 underlined that education is a need for teachers as follows: “*When the distance education system was initiated, computer teachers at the school gave us some information. However, it was not enough for me. More detailed training should be given, and I think it would be much more effective if a distinction was made according to the fields. After all, we need training on how to teach English more effectively remotely.*” Creating a data pool *(f* = *5)* was determined as the second code under this theme. T12 said: “*The preparation of videos by the Ministry that guide teachers can create uniformity in education. A sharing web page can be organized by requesting course videos and documents from all teachers. For example, such a thing can be a guide for teachers who need it. Speaking for myself, I have benefited from EBA and other sites from time to time.*” As the last code, it was suggested that the course contents should be prepared by the teachers *(f* = *2).* T2 suggested: “*On the one hand, there was a concern about teaching the curriculum, but on the other hand, the length of the classes decreased. I think the determination of the content should be left to the teacher so that the subject and time can be adjusted according to the situation of the class. In this way, we can ensure that there is effective learning beyond curriculum concern*” whereas T12 recommended: “*I think the subjects, content and teaching methods in the distance education period should be left to the choice of English teachers. The Ministry should allow us to prepare the plans. The schools are not the same, we cannot progress with stereotypes.*”

## Conclusion, Discussion, and Recommendations

During the fight against the pandemic, which is considered a global tragedy, educators have faced a chaotic test. Even educators who are familiar with the distance education system have had a hard time keeping up with the system during the COVID-19 process. Teachers have several responsibilities in terms of adapting to the rapid change in technology. Foreign language education is at the forefront of the education that should be continued in terms of the development level of specific countries. Therefore, the opinions of English teachers working at secondary school level were taken in order to put forward solutions that can be applied both in macro (policy makers) and micro dimensions (teachers) to the problems that emerged in this extraordinary process.

When the first result of the research is examined, it can be determined that teachers used several applications and made sharings on different platforms in order to increase the efficiency of distance education process. In particular, teachers’ personal e-mail accounts and Whatsapp application have been the most preferred sharing tools. Whatsapp application stands out as the most popular tool used by educators in foreign language teaching (Jasrial, 2018; Tümen-Akyıldız, 2020; Alsaleem, 2021). In addition, by using different virtual platforms, teachers tried to cover the subjects in the curriculum through live classes. In the distance education process, it was seen that the teachers were in contact with both the parents and the students so that the students would not be disconnected from the education process.

The second result of the study revealed that English teachers encountered problems related to technology, students, online classes and planning in the distance education process. Internet shortage and lack of hardware were frequently mentioned technological infrastructure problems in the research. This problem, which affects both the teachers and the students, has a rather negative impact on the distance education system. In the research conducted by Wang et al. (2020), the lack of access to the internet appeared as an important problem related to the distance education process. In addition, it has been revealed that teachers have negative opinions about the process due to insufficient student participation, the lack of interest and motivation of the students toward the class, and the inequality of opportunity in the distance education system. Similarly, there are some research results which suggest that students struggle with problems that affect their achievement and efficiency in distance education, such as the lack of equal conditions in terms of technical infrastructure and opportunities which affect class participation of students (Akyavuz and Çakın, 2020; Kaya, 2020; Balaman and Hanbay Tiryaki, 2021; Stacki et al., 2021).

The third result of the study is that English teachers offer solutions for the problems experienced in the distance education process regarding technology, process and planning. The first point that teachers focus on is the need to provide unlimited internet support to both teachers and students in order to run the system more effectively, to provide students with tablets or computers so that they can access distance education without any problems, and to provide equal opportunities in education. In addition, teachers offered the following suggestions: an effective assessment and evaluation must be included in the distance education system; a hybrid education system must be implemented; distance education seminars should be given to teachers in order to increase their digital competencies, and course content should be developed by teachers. Balaman and Hanbay Tiryaki (2021) recommended that traditional education and distance education could be combined as a result of eliminating the deficiencies identified in the distance education system and fulfilling other needs, especially in terms of infrastructure. In their research, Alves et al. (2021) argued that there is a need for sound education policies and therefore more effective infrastructure investments for distance education studies. In a study evaluating the distance education system from the perspective of different education stakeholders, it is stated that comprehensive training is needed to increase the competence of teachers on the system (Pratama et al., 2020). In addition, studies underline that the planning of a distance education system, which includes a more flexible curriculum, technology-readiness, and developing various learning strategies with the cooperation of stakeholders is critical for a successful education (Rasmitadila et al., 2020).

As a result, although human beings communicate using several different languages, English as a lingua franca is the common language that people use to communicate with each other today. With economic globalization, countries have started to use English as a common language in international trade while promoting and marketing the products they produce. In the upcoming period, as a result of the development of new communication technologies and the pandemic, it may be possible to use distance education learning media for students together with traditional education settings. In this period, it is critical for both the teachers and the students to be informed about distance learning environment, and their experiences play a critical role to ensure that learning occurs in a meaningful way. Since this research aims to reveal the opinions of English teachers about distance education practices, the difficulties they encounter and the solutions they bring to these problems in terms of planning foreign language education policy, it may be important in terms of predicting future innovations for scientists working in the fields of English learning and distance education environments. In addition, when the research is considered with regards to its limitations, various suggestions can be made for researchers. Since this research is limited to secondary schools in Northern Cyprus, it can be possible to see the whole picture by conducting such studies for a larger population of teachers in different school types. In addition, since the research is limited to English teachers, it will be useful to compare and expand the results of studies with other branch teachers with the findings obtained in this study.

With the COVID-19 pandemic, individuals and institutions have had to communicate electronically more than ever before. Therefore, the need to establish a healthy infrastructure has arisen so that distance education and home office working can function properly. There has been an urgent improvement of the digital infrastructure especially in the education sector (Persson, 2020). In addition to the immediate importance of students’ access to information on online platforms, it was also necessary for teachers to organize teaching methods in this system in accordance with pedagogical methods. In addition to the readiness of students and teachers, it is seen that parents and the education policies produced in the online foreign language education process also need to adapt to these new conditions.

## Data Availability Statement

The datasets presented in this study can be found in online repositories. The names of the repository/repositories and accession number(s) can be found in the article/supplementary material.

## Ethics Statement

The studies involving human participants were reviewed and approved by Ethical Committee Board of Ministry of Education of N. Cyprus. The patients/participants provided their written informed consent to participate in this study.

## Author Contributions

Both authors contributed equally to the article writing and conceptualization process.

## Conflict of Interest

The authors declare that the research was conducted in the absence of any commercial or financial relationships that could be construed as a potential conflict of interest.

## Publisher’s Note

All claims expressed in this article are solely those of the authors and do not necessarily represent those of their affiliated organizations, or those of the publisher, the editors and the reviewers. Any product that may be evaluated in this article, or claim that may be made by its manufacturer, is not guaranteed or endorsed by the publisher.
